# Murine norovirus infection does not cause major disruptions in the murine intestinal microbiota

**DOI:** 10.1186/2049-2618-1-7

**Published:** 2013-02-18

**Authors:** Adam M Nelson, Michael D Elftman, Amelia K Pinto, Megan Baldridge, Patrick Hooper, Justin Kuczynski, Joseph F Petrosino, Vincent B Young, Christiane E Wobus

**Affiliations:** 1 Department of Internal Medicine, Division of Pulmonary and Critical Care Medicine, University of Michigan Medical School, Ann Arbor, MI, USA; 2 Department of Internal Medicine, Division of Infectious Diseases, University of Michigan Medical School, Ann Arbor, MI, USA; 3 Department of Microbiology and Immunology, University of Michigan Medical School, 5622 Medical Sciences Bldg. II, 1150 West Medical Center Drive, 48109-5620, Ann Arbor, USA; 4 Department of Medicine, Washington University School of Medicine, St Louis, MO, USA; 5 Department of Pathology and Immunology, Washington University School of Medicine, MO, St Louis, USA; 6 Department of Molecular, Cellular and Developmental Biology, University of Colorado, Boulder, CO, USA; 7 Alkek Center for Metagenomics and Microbiome Research Department of Molecular Virology and Microbiology, Human Genome Sequencing Center, Baylor College of Medicine, Houston, TX, USA

**Keywords:** Microbiome, Murine norovirus, Pyrosequencing

## Abstract

**Background:**

Murine norovirus (MNV) is the most common gastrointestinal pathogen of research mice and can alter research outcomes in biomedical mouse models of inflammatory bowel disease (IBD). Despite indications that an altered microbiota is a risk factor for IBD, the response of the murine intestinal microbiota to MNV infection has not been examined. Microbiota disruption caused by MNV infection could introduce the confounding effects observed in research experiments. Therefore, this study investigated the effects of MNV infection on the intestinal microbiota of wild-type mice.

**Results:**

The composition of the intestinal microbiota was assessed over time in both outbred Swiss Webster and inbred C57BL/6 mice following MNV infection. Mice were infected with both persistent and non-persistent MNV strains and tissue-associated or fecal-associated microbiota was analyzed by 16S rRNA-encoding gene pyrosequencing. Analysis of intestinal bacterial communities in infected mice at the phylum and family level showed no major differences to uninfected controls, both in tissue-associated samples and feces, and also over time following infection, demonstrating that the intestinal microbiota of wild-type mice is highly resistant to disruption following MNV infection.

**Conclusions:**

This is the first study to describe the intestinal microbiota following MNV infection and demonstrates that acute or persistent MNV infection is not associated with major disruptions of microbial communities in Swiss Webster and C57BL/6 mice.

## Background

Noroviruses are highly prevalent, positive-strand RNA viruses that infect the gastrointestinal tract of several mammalian species, including humans [1], mice [2] and cattle [3]. Murine norovirus (MNV) is the most common pathogen in biomedical research mice worldwide [4] with a reported prevalence as high as 64% [5-10]. Following the discovery of MNV-1 in immunocompromised mice [2], additional MNV strains have been isolated from laboratory mice that form one serogroup, despite differences in biological phenotypes [11]. For example, MNV strains MNV-4 and CR6 cause persistent infections and viral genomes are detected in feces and tissues for at least 35 days post infection (DPI), while MNV-1 causes an acute infection and no infectious virus is shed by 7 DPI [2,11,12]. Recently, MNV was also identified in wild rodents [13,14]. Genetically, MNV strains are diverse, exhibiting sequence variation across 23% of the genome [14]. MNV replicates in macrophages (Mϕs) and dendritic cells (DCs) *in vitro*[15] and infectious virus is found *in vivo* in the lamina propria and Peyer’s patches of the intestine, and mesenteric lymph nodes [16,17]. MNV-infected mice show varying degrees of clinical disease. Wild-type mice show no overt signs of disease during MNV infection [16,18,19], while some immunocompromised mouse strains succumb to lethal infection [2]. MNV can also change research outcomes in mouse models. MNV-4 alters disease progression in a mouse model of bacterially-induced inflammatory bowel disease (IBD) [20], and infection with the MNV strain CR6 induced intestinal inflammation resembling Crohn’s disease in mice with a genetically susceptible background (*ATG16L1*) [21]. In the latter case, the observed pathologies were in part dependent on an intact microbiota. Disruption of the microbiota is common following bacterial infection of the gastrointestinal tract and is associated with diseases such as IBD and post-infectious irritable bowel syndrome (see, for example, [22,23]). This raised the question of whether MNV infection changes the murine intestinal microbiota. Since this is currently unknown, our study sought to determine the extent to which MNV infection disrupts the bacterial community of the murine intestine.

Inbred and outbred immunocompetent mice were infected with persistent or non-persistent strains of MNV (MNV-1, MNV-4, CR6). In independent, but complementary experiments conducted at two institutions, the composition of fecal-associated or tissue-associated microbial communities was determined at various times post infection by pyrosequence analysis of the bacterial 16S rRNA-encoding gene amplicons. No major alterations in the murine intestinal microbiota were detected at the phylum and family levels in both Swiss Webster and C57BL/6 mouse strains after MNV infection. These results suggest that the intestinal microbiome of immunocompetent inbred and outbred mouse models are resistant to MNV infection. Further studies are needed to determine the ability of microbial communities to resist MNV disruption in immunocompromised mice.

## Methods

### Virus

MNV strain MNV-1 (GV/MNV1/2002/USA), plaque-isolate CW3 (GenBank accession no. EF014462) [11] was used at passage 6. MNV strain MNV-4 (GV/MNV4/2005/US; GenBank accession no. DQ223043), a field isolate [12], was used at passage 3. MNV strain CR6 (GV/CR6/2005/US; GenBank accession no. EU004676) was plaque purified three times as previously described for MNV-1 [19] and used at passage 3. Virus lysates were concentrated by ultracentrifugation and resuspended in phosphate-buffered saline (PBS) as described previously [24]. Mock-infected lysates were prepared in a similar manner and used as controls.

### Swiss Webster (outbred strain) infection and sample collection

Swiss Webster infection studies were performed as two separate experiments. For both experiments, 7-week-old male outbred Swiss Webster mice were purchased from Charles River Laboratories. All mice were seronegative for MNV at the onset of the experiments. Mice for each experiment were purchased in separate batches and are thus described separately. Mice were housed at the University of Michigan animal facilities. Animal studies were performed in accordance with local and federal guidelines as outlined in the ‘Guide for the Care and Use of Laboratory Animals’ of the National Institutes of Health. The protocol was approved by the University of Michigan Committee on Use and Care of Animals (UCUCA no.: 09710). The first experiment used nine replicate cages, with three mice cohoused per cage and per treatment group. Mice were infected orally with 1 × 10^7^ plaque forming units (pfu) of MNV-1 (n = 27) in a volume of 25 μl PBS, or mock lysate (n = 27) as a control. Tissue samples from the distal ileum, cecum, and colon were collected on days 7, 28, and 56 post infection. Intestinal contents were removed, and half of the cecum and 1 cm pieces of ileum and colon were snap frozen and stored at −80°C.

In the second experiment a total of six replicate cages with three mice cohoused per cage were used per treatment group. Mice were orally infected with 3 × 10^6^ pfu of either MNV-1 (n = 18) or MNV-4 (n = 18) in a volume of 30 μl PBS or mock lysate (n = 18) as a control. Tissue samples from the distal ileum, cecum and colon were collected on days 1 and 3 post infection as described above. Infection was verified by measuring viral shedding in fecal pellets collected at the time of tissue harvest by Charles River Laboratories Research Animal Diagnostic Services (Wilmington, MA, USA) using quantitative reverse transcription real-time polymerase chain reaction (qRT-PCR) as described previously [11].

### DNA extraction (Swiss Webster)

DNA was extracted from murine intestinal tissues using the Roche MagNA Pure Compact system (Roche Diagnostics GmbH, Mannheim, Germany). Tissue samples were suspended in a mixture of Roche Bacterial Lysis Buffer (Roche Diagnostics GmbH, Mannheim, Germany) and sterile PBS (pH 7.4, Invitrogen, Carlsbad, CA, USA). Tissue was disrupted by bead-beating for 1 minute, followed by treatment with 50 μl of proteinase K for 10 minutes at 65°C. Samples were further disrupted by 1 minute of additional bead beating, before heat inactivation at 95°C for 10 minutes. Additional sample processing was performed on the MagNA pure compact system according to the Roche MagNA Pure Nucleic Acid Isolation Kit I protocol (Roche Diagnostics GmbH, Mannheim, Germany). Extracted DNA was quantified on a NanoDrop 1000 spectrophotometer (NanoDrop, Wilmington, DE, USA) and stored at −20°C.

### Pyrosequencing (Swiss Webster)

Tissue-derived DNA samples were submitted for 16S rRNA gene amplification and pyrosequencing in two separate batches at the Human Genome Sequencing Center at Baylor University College of Medicine in Houston, TX, USA and the University of Michigan DNA Sequencing Core in Ann Arbor, MI, USA. The V3 to V5 region of the 16S rRNA gene was amplified following the Broad HMP protocol (HMP MDG Default Protocol v4.2), which can be found at: http://www.hmpdacc.org/doc/16S_Sequencing_SOP_4.2.2.pdf.

Amplified PCR products were checked for quality on a 2% agarose gel for visual verification, and each sample was individually quantified using the Quant-It PicoGreen dsDNA kit (Molecular Probes, Eugene, OR, USA). Each sample was diluted to normalize concentrations before pooling. The pooled sample was then checked on a Bioanalyzer 2100 machine (Agilent, Santa Clara, CA, USA) using a DNA1000 lab chip (Agilent) to verify sample purity prior to amplification by emulsion PCR and pyrosequencing.

### C57BL/6 (inbred strain) infection and sample collection

In an independent experiment performed in parallel, 7-week-old male C57BL/6 mice (n = 20), five siblings from four sets of identified parents, were used. The mice were bred at Washington University School of Medicine under specific pathogen-free conditions [21] in accordance with all Federal and University policies. Mice were divided into four treatment groups with one to two mice from each sibling group placed into each treatment group, including untreated control (n = 5), mock infected control (n = 5), MNV-1 infected (n = 5), and CR6 infected (n = 5). All mice were singly housed for the duration of the experiment. All groups except the untreated control mice were given either sterile PBS (25 μl) for mock infection, or orally infected using 3 × 10^7^ pfu of CR6. Feces were collected from each mouse on days 0, 1, 2, 4, 8, 28 and 42 following infection. All mice were seronegative at the initiation of the experiments and as expected only the MNV-infected mice were seropositive at the conclusion of the experiment.

### DNA extraction (C57BL/6)

Fresh fecal samples were harvested into sterile screw-top 2 ml tubes containing 500 ml of 0.1 mm zirconia/silica beads (BioSpec, Bartlesville, OK, USA) and stored at −80°C. DNA was isolated by phenol-chloroform extraction and cleaned up using the AMpure (Agencourt, Beckman Coulter, Inc., Brea, CA, USA) system according to the manufacturer’s protocol. The DNA was then diluted to a concentration of 10 to 100 ng/μl.

### Pyrosequencing (C57BL/6)

Each DNA sample was setup in triplicate and pooled at the end of each PCR run to avoid founder effects. The primers used were described previously [25] and amplified the V1 to V2 region of the 16S rRNA encoding gene, with a universal forward primer and a reverse primer with the addition of a linker and an eight base pair barcode (27F-TCAGAGTTTGATCCTGGCTCAG, 338R-NNNNNNNNCATGCTGCCTCCCGTAGGAGT). The DNA was amplified using 5 Prime Hotmastermix (5Prime Inc., Gaithersburg, MD, USA) with cycling conditions identical to those published previously [26]. The pooled PCR products were run on an agarose gel to confirm the generation of a 300 bp product prior to the submission to the Genome Sequencing Center (GSC) at Washington University, St Louis, USA, for emulsion PCR amplification and 454 Pyrosequencing.

### Pyrosequencing data processing and analysis

Analysis of all 454 pyrosequencing data was performed using mothur (version 1.20) [27]. The standard operating procedure instructions for pyrosequencing data processing on the mothur website were followed and can be found at: http://www.mothur.org/wiki/Schloss_SOP.

Pyrosequencing data for both Swiss Webster mouse experiments were analyzed together, while data from C57BL/6 mice were analyzed separately. Both data sets were then processed analogously. First, mothur was used for assigning operational taxonomic units (OTUs), community structure comparisons and classification of 16S rRNA sequences. Classifications were determined by comparing sequences to the Ribosomal Database Project (RDP) (Michigan State University, East Lansing, MI, USA) [28]. Next, sequences were trimmed to remove any ambiguous base calls, those with more than 8 homopolymers, and those with an average quality score below 35 in a window of 50 nucleotides. After trimming, sequences were filtered based on size, and all reads less than 194 nucleotides (Swiss Webster data set) or 219 nucleotides (C57BL/6 data set) were removed. Filtering thresholds were determined based upon alignment with the silva alignment database (http://arb-silva.de/) for each data set. Specifically, during the column alignment, the vertical setting in mothur was set to ‘true’ to remove any column containing gap characters, and the trump setting was set to ‘.’, so any column containing a blank nucleotide was also removed during filtering. Additionally, reads with one or more ambiguous calls were also removed. Only sequences containing the reverse primer were used in the analysis.

OTUs were assigned to sequences based on 97% sequence identity prior to RDP classification. Classified OTUs were used to determine the relative abundance of bacterial phyla in each sample and for statistical comparisons between samples.

Principal coordinates analysis (PCoA) was used to assess community similarity among samples by representing the relative abundance of OTUs in each community using two different analyses. First, a Yue and Clayton-based distance matrix [29], which measures community structure by incorporating both membership and abundance, was generated. Second, OTU data was analyzed using PCoA via the Jaccard dissimilary index [30], which measures only membership. These distances were displayed visually in two-dimensional space in PCoA plots. Communities clustered similarly by tissue site, but not by infection status or time, using both Yue and Clayton and Jaccard indices, thus the latter analysis is not shown.

Non-metric multidimensional scaling (NMDS) was used to assess community similarity. NMDS-based ordinations served as a comparator to PCoA to view the data with reduced distortion due to the horseshoe effect seen in PCoA. NMDS values were calculated using the NMDS command in mothur, using all default parameters, and those values were displayed visually in two-dimensional space.

Phylotype analysis was performed using relative abundance values for comparison at both the phylum and family taxonomic levels once relative abundance measurements were averaged for replicate samples in each tissue and treatment group.

DNA sequences are publically available via MG-RAST. Swiss Webster mice are available at: http://metagenomics.anl.gov/linkin.cgi?project=3128. Barcodes for individual Swiss Webster sequences are included in Additional file 1: Table S1. C57BL/6 mice sequences are available at http://metagenomics.anl.gov/linkin.cgi?metagenome=4506745.3. Barcodes for individual C57BL/6 samples are included in Additional file 2: Table S2.

Neither study design measured the bacterial density, so it remains unknown whether MNV infection can raise or lower overall bacterial densities in the intestine.

### Statistical analysis

The relative abundance values of specific bacterial phyla were compared using both the Kruskal-Wallis test of variance and the Mann–Whitney *U* test. Metastats [31], a statistical test used to determine differentially abundant features, was used within the mothur bioinformatics program to determine significant differences (at *P* = 0.05) of specific OTUs in treatment groups based on 3% OTU definitions. All other statistical analyses were performed using GraphPad Prism version 5 (GraphPad Software, San Diego, CA, USA). Measurements of community richness (Chao and ACE) and diversity (Shannon and inverse Simpson) were calculated using mothur, and were based on a 3% OTU definition. ACE richness estimates were based on an OTU with ten or more individuals in it being considered abundant. The number of total OTUs was determined by the sobs calculator in the summary.single command within mothur. It was also based on a 3% OTU definition. These values, and richness and diversity estimates are included in Additional file 1: Table S1 and Additional file 2: Table S2.

When comparing phylotype abundance in either Swiss Webster or C57BL/6 mice data, individual timepoints were combined so comparisons could be made between treatments. This was done because the communities were stable over time, both in Swiss Webster and C57BL/6 mice, and showed little variation throughout the course of the experiment. Specifically, all times were combined in order to compare relative abundance of specific phylotypes between each treatment group. Furthermore, times were also combined for comparison at the OTU level between each treatment group.

## Results

### The intestinal microbiome of Swiss Webster mice is not disrupted by MNV infection

**Figure 1:**
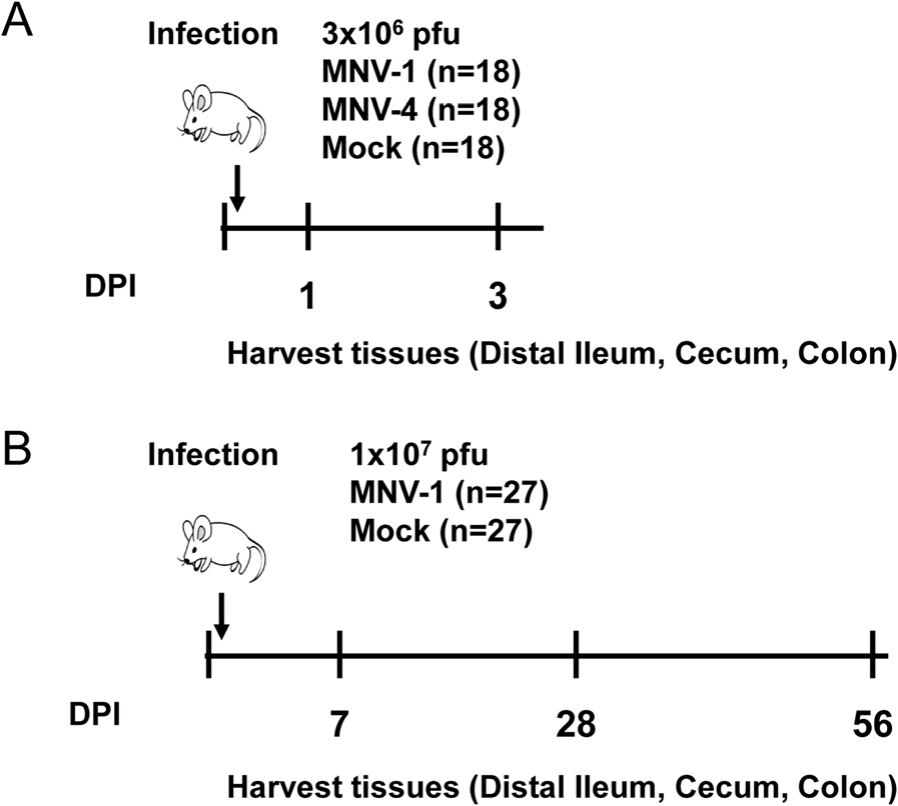
**Mouse infection schemata for Swiss Webster mice.** Swiss Webster mice were infected orally in two separate experiments to determine effects on intestinal tissue-associated microbial communities. (**A**) Mice were infected orally with two murine norovirus (MNV) strains, MNV-1 and MNV-4, and compared to a mock-infected control. At days 1 and 3 following infection intestinal tissues were harvested. (**B**) Mice were infected orally with MNV-1 or mock lysate and tissues harvested on days 7, 28, and 56 post infection. DPI = days post infection; pfu = plaque forming units.

To determine whether MNV infection causes large-scale changes in the intestinal microbiota of outbred Swiss Webster mice, two separate experiments were set up (Figure 1). Short-term alterations in the intestinal microbiota in wild-type mice were analyzed in Swiss Webster mice 1 and 3 days following infection with MNV strains MNV-1 and MNV-4 or mock lysate (Figure 1A). These early timepoints represent the peak of viral infection [2,16]. MNV-4 was included because this strain increased the severity of disease in a mouse model of IBD [20] and a change in the microbial community is a risk factor for development of IBD [32]. Long-term alterations in the intestinal microbiota after clearance of MNV infection were analyzed in mice infected with MNV-1, which causes an acute infection [2,11,12], or mock lysate for 7, 28, and 56 days (Figure 1B). In both experiments, DNA was extracted from distal ilea, ceca and colons of animals for 16S rRNA gene amplification and barcoded 454 pyrosequencing. A total of 282 samples were amplified (Additional file 3: Table S3) generating 1,473,332 total high-quality 16S reads that were used for community analysis. A summary of alpha diversity measurements for these samples is shown in Additional file 1: Table S1.

Measurements of community richness and diversity were compared between treatment groups (Additional file 4: Table S4). Early (days 1 and 3 post infection) and late (days 7, 28, and 56 post infection) timepoints were combined because measurements of diversity and richness were consistent over time. No differences were seen using the Kruskal-Wallis test of variance between infected and control samples within a tissue source for all comparisons except for the late timepoints with Chao richness measurements in the distal ileum and with Shannon diversity in the colon (highlighted in Additional file 4: Table S4). To determine if these significant differences could be influenced by combining sampling times, specific comparisons for each timepoint were performed separately using the Mann–Whitney *U* test. No significant differences were seen in these comparisons. Specifically, MNV-1 infected mice versus mock controls were compared separately at day 7 post infection (*P* = 0.3450), day 28 post infection (*P* = 0.2319), and day 56 post infection (*P* = 0.7242) for the Chao richness in the distal ileum. Shannon diversity was not different in colon samples at post-infection days 7 (*P* = 0.6607), 28 (*P* = 0.0712), and 56 (*P* = 0.6126). Therefore, no evidence was found that either MNV-1 or MNV-4 infection alters richness or diversity measurements compared to mock-infected controls in Swiss Webster mice in any tissue examined.

**Figure 2:**
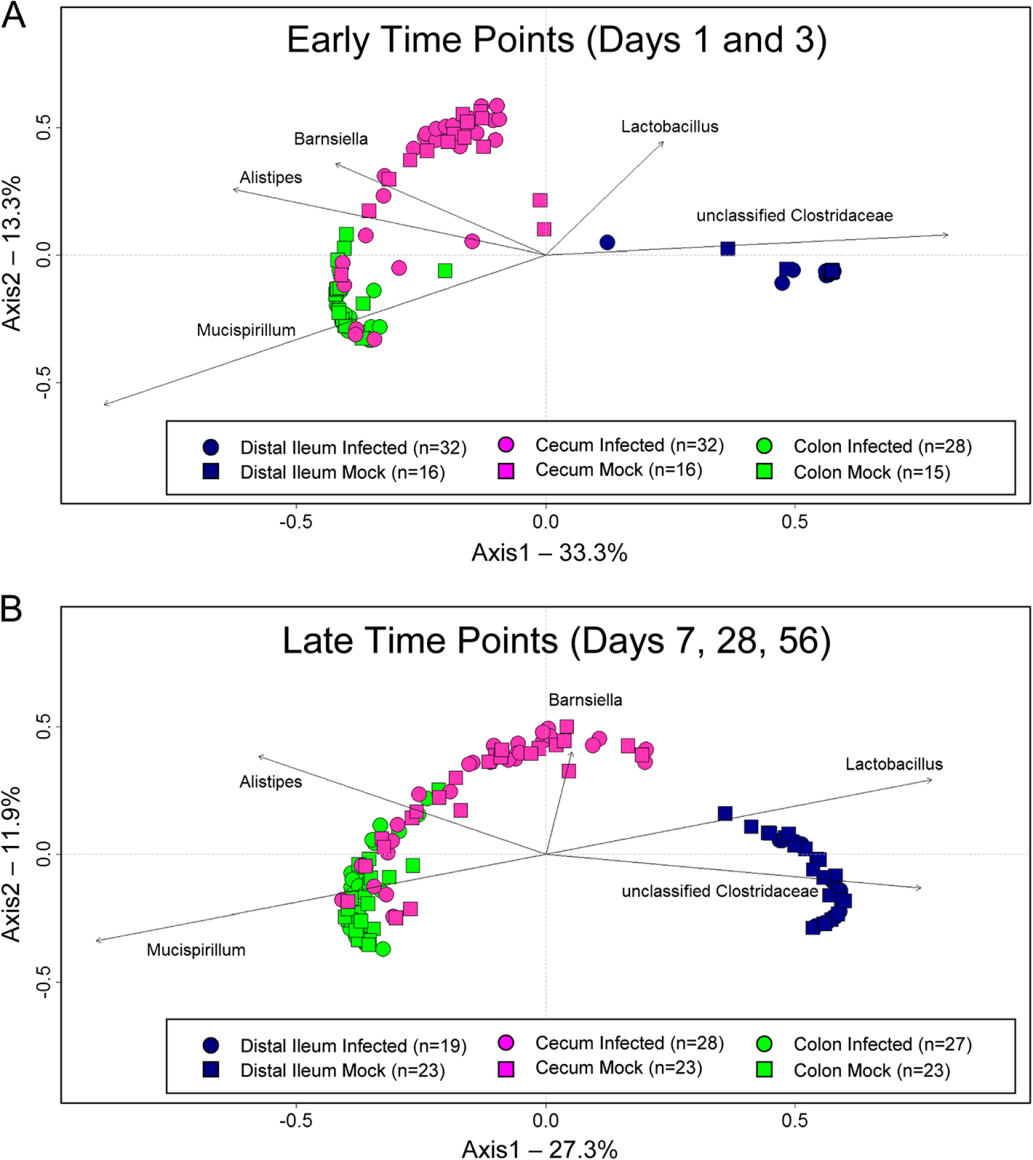
**Communities in Swiss Webster mice differ by tissue site but not by infection status.** This principal coordinates analysis plot represents the relative operational taxonomic unit (OTU) abundance in Swiss Webster mice at a 3% definition level, and was generated using a Yue and Clayton distance matrix. (**A**) Community structure at early timepoints, days 1 and 3 post infection. (**B**) Community structure at late timepoints, days 7, 28, and 56 post infection. Each murine intestinal community is represented by a symbol. Each symbol is colored to represent the tissue location of the community. Arrows represent the direction where the most frequently detected OTUs influence the location of each sample. All times were combined for each experiment.

To visualize the relationship among communities, principal coordinates analysis (PCoA) was performed on the OTU diversity data (Figure 2). Similar communities are located close to one another in the plot, while divergent communities are located further apart. At both early (Figure 2A) and late timepoints (Figure 2B), communities were distinguished according to the tissue site using both Yue and Clayton (Figure 2) and Jaccard indices (data not shown). The most divergent communities were found in the distal ileum, which were dominated by unclassified *Clostridaceae*. The cecum was largely defined by *Barnsiella*, while the colon was mostly predominated by *Mucispirillum*. However, there was no apparent clustering that separated the communities between infected and uninfected mice, and the community structure over the course of the experiment was very consistent and driven by a predominance of *Barnsiella*, *Alistipes*, *Mucispirillum*, and unclassified *Clostridaceae*. Thus, infection did not alter the bacterial community structure in tissue-associated communities, suggesting that MNV infection had no effect (either direct or indirect) on the bacterial microbiota. In addition, an ordination was performed using non-metric multidimensional scaling on this data to account for the spatial interrelationships of the data points (Additional file 5: Figure S1). Because of similarities in community structure over time, these are shown with all times combined in experiments from either the early or late timepoints. This analysis confirmed the observed differences in communities by tissue site and lack thereof by infection status.

**Figure 3:**
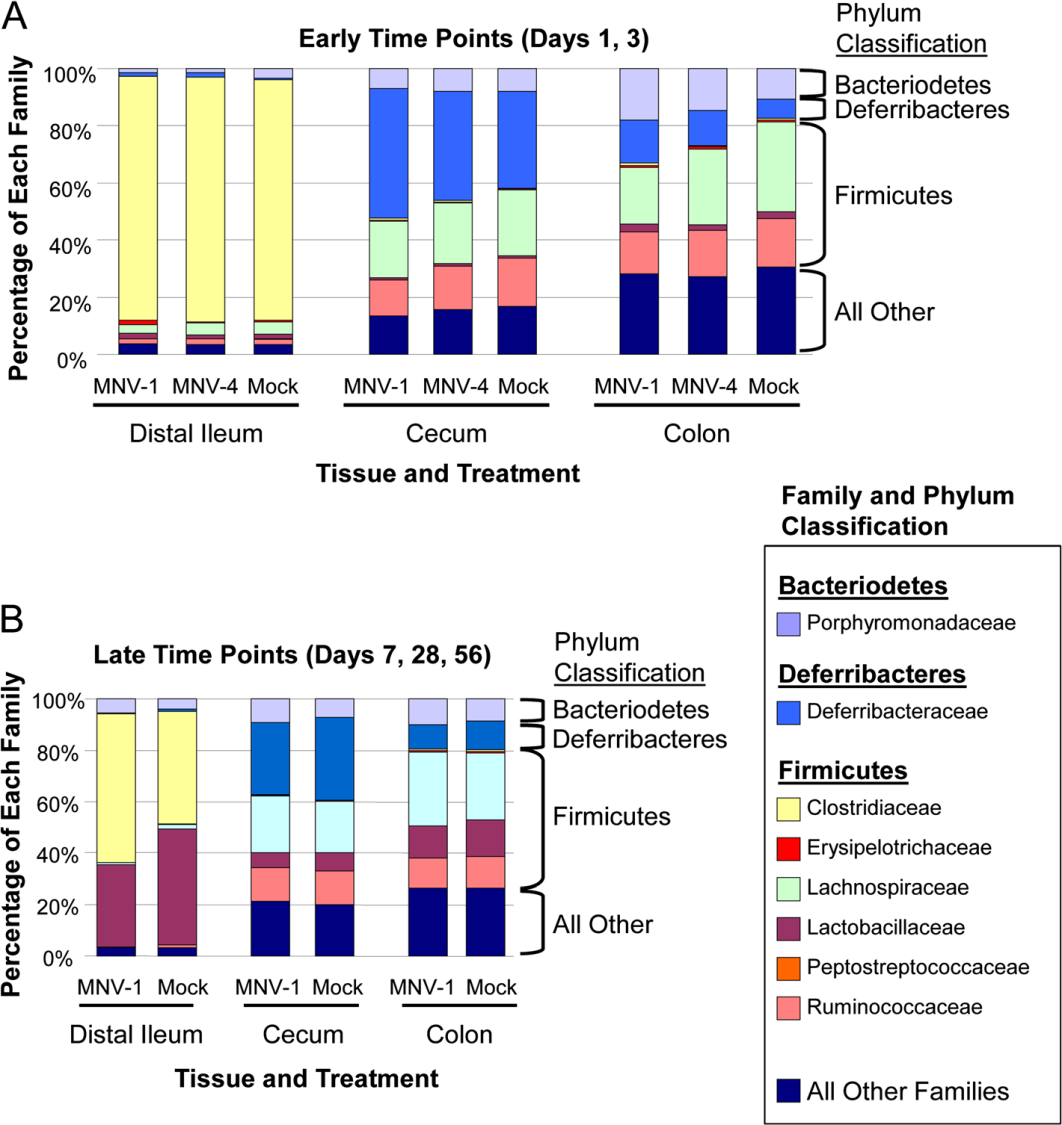
**Family level diversity comparisons reveal significant differences by tissue, but not by infection status, in Swiss Webster mice.** Comparisons of bacterial family and phyla are shown across tissues and treatment groups. Relative abundance values for each classification were calculated for individual replicates, then averaged for comparisons. All timepoints for each experiment were combined. Mock = mock-infected control.

Sequences of representative OTUs were compared to known sequences in the database to determine the bacterial classification at the phylum and family levels (Figure 3). Microbial communities were dominated by *Bacteriodetes*, *Deferribacteres*, and *Firmicutes*. Relative abundance of each major phyla were compared between infected and control groups. Significant differences were only detected at early timepoints (days 1 and 3) in the cecum for *Deferribacteres* (*P* = 0.0135), and in the colon for *Bacteriodetes* (*P* = 0.0073) and *Firmicutes* (*P* = 0.0032), when comparing MNV-1 to mock-infected mice using the Mann–Whitney *U* test. There were no significant differences in the detection of these sample phyla between infected and control groups in the same tissue at later timepoints. In addition, the strain of MNV used also did not affect phylum level detection in any tissue examined for either early or late timepoints. The major difference seen between communities was based upon the tissue source, with cecum and colon being more similar than distal ileum. When compared broadly, the levels of *Bacteriodetes*, *Firmicutes*, and *Deferribacteres* were significantly different in the distal ileum compared to either the cecum or colon (*P* <0.0001) as measured by the Kruskal-Wallis test.

To determine whether MNV infection caused major alterations in bacterial communities at the genus level, the ten most frequently detected genera were compared for early timepoints (MNV-1 vs mock, MNV-4 vs mock, and MNV-1 vs MNV-4) and late timepoints (MNV-1 vs mock) using the Kruskal-Wallis test with Dunn’s multiple comparisons. No comparison of infected versus control in the same tissue was significantly different at either early or late timepoints. Furthermore, no differences were detected when comparing MNV-1 versus MNV-4 infected animals within each tissue. Next, the 25 most abundant OTUs in Swiss Webster mice were examined for differences between infected and uninfected groups by metastats. Only 5 of 225 comparisons had significantly different detection (highlighted in Additional file 6: Table S5). In both of these analyses all timepoints were combined because communities were consistent over time. These data indicate that a small fraction of specific OTUs had differences in relative abundance. However, the biological significance of these differences is unclear.

In summary, while finer-scale comparisons on the family, genus, or OTU level indicated a few minor alterations of unclear biologic significance between infected and control communities, no large-scale alterations in the bacterial microbiota were seen following MNV infection. Analysis at the phylum level demonstrated major differences in community composition by tissue site and a largely unchanged intestinal tissue-associated microbiota following MNV infection in Swiss Webster mice.

### The microbiome of C57BL/6 mice is resistant to disruption by MNV

**Figure 4:**
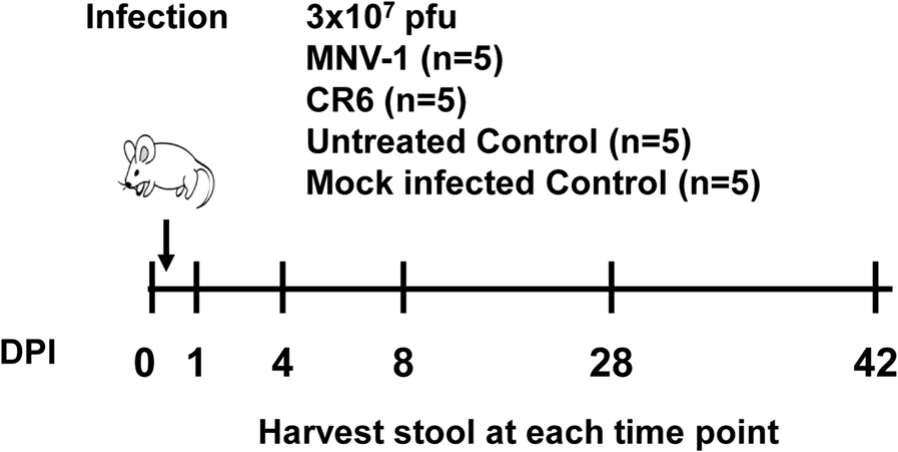
**Mouse infection schemata for C57BL/6 mice.** C57BL/6 mice were orally infected with either murine norovirus (MNV)-1, CR6, mock lysate, or left untreated, and fecal samples were collected at indicated timepoints following infection. DPI = days post infection; pfu = plaque forming units.

**Figure 5:**
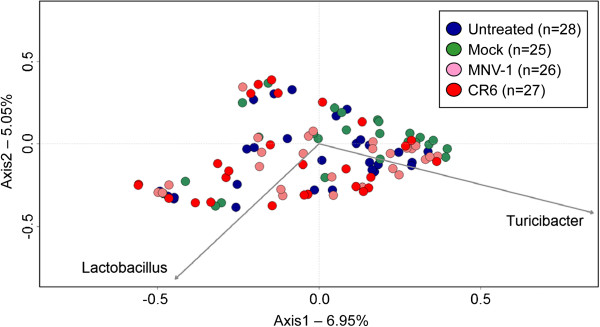
**The intestinal microbiota of C57BL/6 mice is not altered by murine norovirus (MNV) infection.** This principal coordinates analysis plot was generated using relative operational taxonomic unit (OTU) abundance data in a Yue and Clayton diversity metric. Each symbol represents the microbiota of a single stool-associated community from mice infected with either MNV-1 or CR6, or from mock lysate-infected or untreated controls. In this plot, all timepoints for each treatment group were combined. The arrows indicate the most frequent OTUs detected in samples and the direction they influence where samples are indicated on the plot.

**Figure 6:**
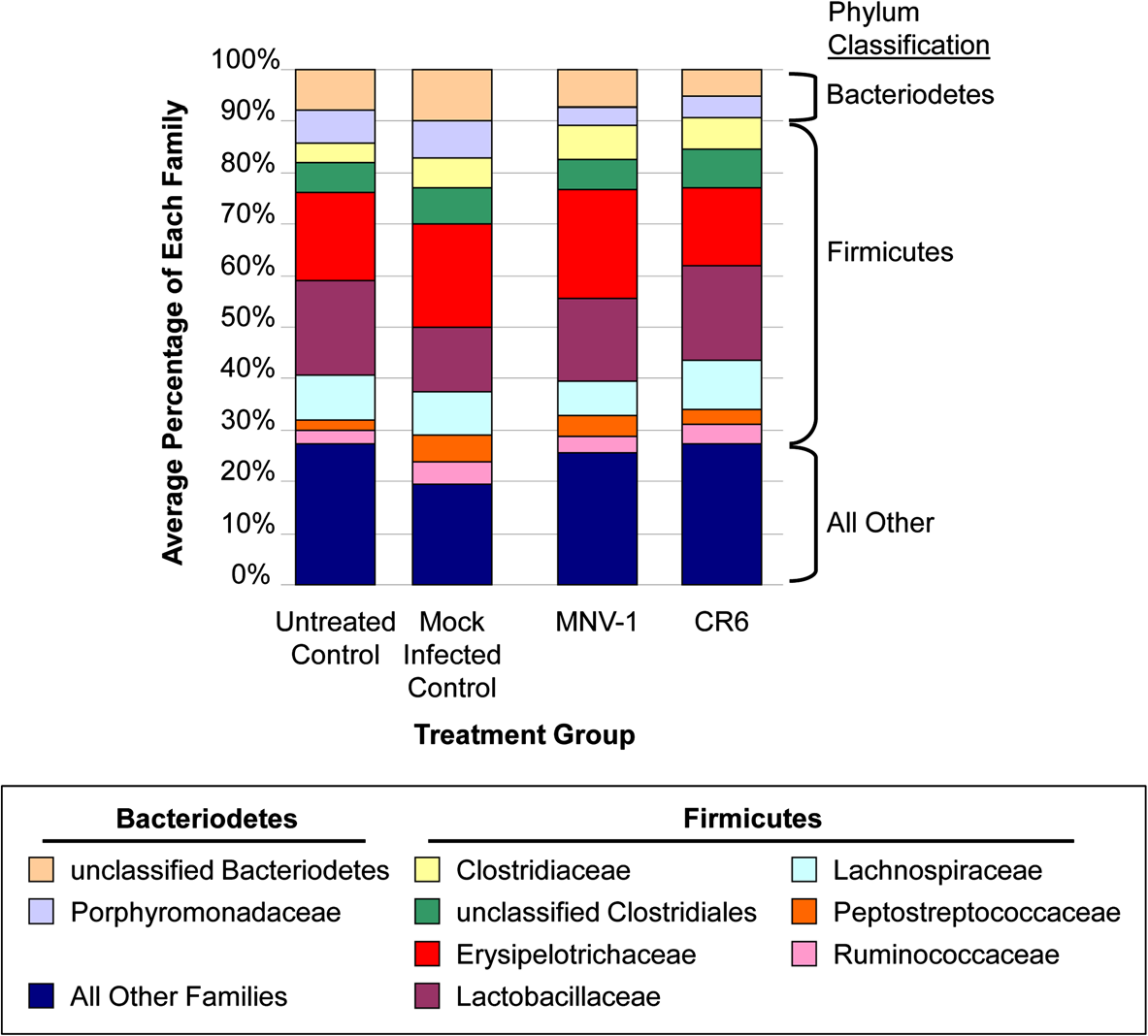
**Phylotype abundance comparisons reveal no significant differences by treatment in C57BL/6 mice.** Comparison of the average relative abundance of family level classification across each treatment group. For each replicate, the relative abundance of each family was calculated, then averaged for each group. All timepoints were combined for comparison.

To determine whether MNV infection was able to alter the fecal microbial community, a separate, but complementary experiment was performed in parallel. In this experiment, C57BL/6 mice were infected with MNV-1 and CR6 (Figure 4). CR6 was included since this strain, but not MNV-1, induces a Crohn’s disease-like phenotype in a microbiota-dependent manner in genetically susceptible Atg16L1^HM^ mice (on the C57BL/6 background) [21]. As controls, mice were either mock infected or left untreated. Feces were collected at multiple timepoints for DNA extraction, 16S rRNA-encoding gene amplification, and barcoded pyrosequencing. A total of 106 samples were sequenced (Additional file 7: Table S6), generating 149,938 high-quality reads for community analysis. A summary of alpha diversity measurements for C57BL/6 mice is shown in Additional file 2: Table S2. There were no significant differences in Shannon diversity (*P* = 0.7158), inverse Simpson (*P* = 0.7546), or Chao richness (*P* = 0.9955) when comparing means between treatment groups using the Kruskal-Wallis test. Because groups were stable over time, all timepoints were combined for these comparisons. As above, the community structure was compared using PCoA generated from OTU community composition (Figure 5). No apparent clustering was observed between MNV-infected and uninfected communities. Infection with either MNV-1 or CR6 strains also caused no alterations in the microbiota, and no differences between infected groups were evident. Phylotype comparisons (Figure 6) further verified that infection did not alter the intestinal communities on the phylum and family taxonomic level. No significant changes were seen in the relative abundance of the most common phylum and family level classifications when comparing infected and uninfected mice using the Kruskal-Wallis test. Specifically, the phylum level comparison included *Bacteriodetes* (*P* = 0.0693) and *Firmicutes* (*P* = 0.3945), while the family-level comparisons of the nine most frequently classified families seen in Figure 6 included unclassified *Bacteriodetes* (*P* = 0.1280), *Porphyromonadaceae* (*P* = 0.5811), *Clostridiaceae* (*P* = 0.2412), unclassified *Clostridiales* (*P* = 0.5354), *Erysipelotrichaceae* (*P* = 0.3612), *Lactobacillaceae* (*P* = 0.2177), *Lachnospiraceae* (*P* = 0.4567), *Peptostreptococcaceae* (*P* = 0.1150), and *Ruminococcaceae* (*P* = 0.1511). At the genus level, there were also no significant differences when comparing the ten most frequently detected genera between all treatment groups using the Kruskal-Wallis test (data not shown). Furthermore, there were no significant differences in the numbers of the top 25 most frequent OTUs detected when comparing both MNV-1 or CR6 versus either the untreated or mock infected controls, as determined by metastats (Additional file 8: Table S7) [31]. Taken together, these data demonstrated that the fecal microbiota of C57BL/6 mice is similar between MNV-1-infected and CR6-infected groups and between infected and uninfected animals. Furthermore, these data are in agreement with findings from intestinal tissues of Swiss Webster mice, which similarly indicated that the microbial communities are not significantly altered by MNV infection.

## Discussion

The microbiome plays an important role in health and disease, including IBD [33,34]. Some MNV strains cause inflammatory pathologies in research models of IBD [20,21]. If MNV infection can alter the murine intestinal microbiota, this may indirectly change the host response and cause such confounding effects in experiments. Therefore, the goal of the current study was to determine whether MNV infection altered the fecal-associated or tissue-associated microbiota of outbred or inbred wild-type mice using pyrosequencing of the bacterial 16S rRNA-encoding gene.

This study used two separate but complementary experimental designs to examine the response of the murine intestinal microbiota to MNV infection. The first study addressed the impact of MNV infection on tissue-associated bacterial communities in Swiss Webster mice, while the second study examined fecal bacterial communities in C57BL/6 mice. Results from both experiments demonstrated that the microbial communities in intestinal tissue or feces of Swiss Webster or C57BL/6 mouse strains did not exhibit major alterations following MNV infection. This was true even for the distal ileum, the site of highest MNV-1 replication [16], indicating that viral replication in intestinal tissues of wild-type mice does not lead to major, local disruptions of the microbiota. In analogy, a recent study demonstrated that MNV titers also are not predictive of intestinal pathology in wild-type mice [35]. Furthermore, no large-scale changes in the intestinal microbiota were seen in the setting of three different MNV strains, including CR6 and MNV-4. These strains were hypothesized to cause disruptions in the microbiota because they were previously implicated in phenotypic alterations of IBD, a disease linked to the dysbiosis of the intestinal microbiome, and caused a Crohn’s-like disease in a microbiota-dependent manner [20,21]. One major difference between the studies is that the disease phenotypes were observed in genetically altered mouse strains, while the current study was performed in wild-type mice. Since mouse genotype is known to influence the make-up of the bacterial community in the intestine [36], future studies are needed to address whether mouse genotype determines the ability of MNV infection to alter the intestinal microbiota.

Furthermore, microbiota disruption following MNV infection may result indirectly from changes in host physiology (for example, altered gastrointestinal transit time or increased fluid secretion) due to infection. In case of the related human norovirus, we recently demonstrated disruption of the intestinal microbiota in nearly one in five people with symptomatic human norovirus infection [37]. However, no overt clinical disease was observed in wild-type mice in the current study or in previous studies [16,38]. Since MNV infection can cause clinical disease in immunocompromised mouse models, future studies are needed to address whether immune status and/or clinical disease severity contribute to the ability of MNV to alter the microbiota [20,39].

Additional factors that may be critical in determining whether enteric virus infections alter the intestinal microbiota could be virus dose or the site of viral replication. We think it is unlikely that virus dose affected the study outcome since the doses used in this study are similar to the largest dose previously reported (that is, 3 × 10^7^ pfu/mouse) [11]. Instead, we hypothesize that the site of virus replication may be an important determinant of whether a given enteric virus infection disrupts the intestinal bacterial community. MNV replicates in macrophages and dendritic cells but not in intestinal epithelial cells [15,19]. Thus, infected cells are not in contact with the intestinal bacterial microbiota, potentially resulting in the observed lack of major alterations in bacterial community and structure.

The results in the current study did demonstrate differences in intestinal communities from different tissue sites in Swiss Webster mice independent of infection status, with the bacterial community of the distal ileum being the most divergent compared to cecum and colon, which were mostly overlapping. This is similar to previous studies in which cecum and colon, but not ileum, were also the most similar sites when comparing bacterial communities in wild-type versus MyD88-deficient C57BL/6 mice [36]. Similarly, spatial differences are observed in humans [40] and C57BL/6 gnotobiotic mice transplanted with human fecal bacteria [41]. Therefore, our study agrees with previous research showing the murine intestine harbors different bacterial communities at different sites along the intestine.

## Conclusions

The current study demonstrated that MNV infection with MNV strains causing acute (MNV-1) or persistent (MNV-4 and CR6) infection does not disrupt the tissue-associated or fecal-associated bacterial communities of two wild-type mouse strains, outbred Swiss Webster and inbred C57BL/6. This is the first study to use pyrosequencing to describe the intestinal community dynamics following MNV infection, and the first to demonstrate that the intestinal microbiota of wild-type mice is largely resistant to MNV infection. These results suggest that microbiome research using wild-type mice may not be affected by MNV infection. However, further studies are needed to determine the ability of MNV infection to alter microbial communities in mice with different genetic backgrounds.

## Competing interests

The authors declare that they have no competing interests.

## Authors’ contributions

Conceived and designed the experiments: AMN, MDE, AKP, VBY, CEW. Performed the experiments: AMN, MDE, AKP, PH, MB. Analyzed the data: AMN, MDE, JK, VBY, CEW. Contributed reagents/materials/analysis tools: AMN, MDE, JFP, JK, MB. Wrote the paper: AMN, MDE, AKP, VBY, CEW. All authors read and approved the final manuscript.

## Supplementary Material

Additional file 1: Table S1 Alpha diversity measurements from pyrosequencing of the Swiss Webster tissue-associated microbiota. Click here for file

Additional file 2: Table S2 Alpha diversity measurements from pyrosequencing of the C57BL/6 tissue-associated microbiota. Click here for file

Additional file 3: Table S3 Summary of the number of tissue-associated DNA samples from Swiss Webster mice used for pyrosequencing. Click here for file

Additional file 4: Table S4 Comparisons in Swiss Webster mice of richness and diversity variance between treatment groups using the Kruskal-Wallis test. Click here for file

Additional file 5: Figure S1 Non-metric multidimensional scaling confirms communities are different by tissue site, but not by infection status, in Swiss Webster mice. This non-metric multidimensional scaling plot represents the relative operational taxonomic unit (OTU) abundance in Swiss Webster mice at a 3% definition level. (**A**) Community structure at early timepoints, 1 and 3 days post infection (DPI). (**B**) Community structure at late timepoints, 7, 28, and 56 DPI. Each murine intestinal community is represented by a symbol. Each symbol is colored to represent the tissue location of the community. All times were combined for each experiment. Click here for file

Additional file 6: Table S5 Comparison of operational taxonomic unit (OTU) abundance in the top 25 most frequent OTUs in Swiss Webster mice between treatment groups using Metastats. Click here for file

Additional file 7: Table S6 Summary of the number of stool-associated DNA samples from C57BL/6 mice used for pyrosequencing. Click here for file

Additional file 8: Table S7 Comparison of operational taxonomic unit (OTU) abundance in the top 25 most frequent OTUs in C57BL/6 mice between treatment groups using Metastats. Click here for file
