# Supplementary figures and images for: Murine norovirus infection does not cause major disruptions in the murine intestinal microbiota
Source: Microbiome. 2013 Feb 18;1:7. doi: 10.1186/2049-2618-1-7 (PMC4177540; doi:10.1186/2049-2618-1-7)

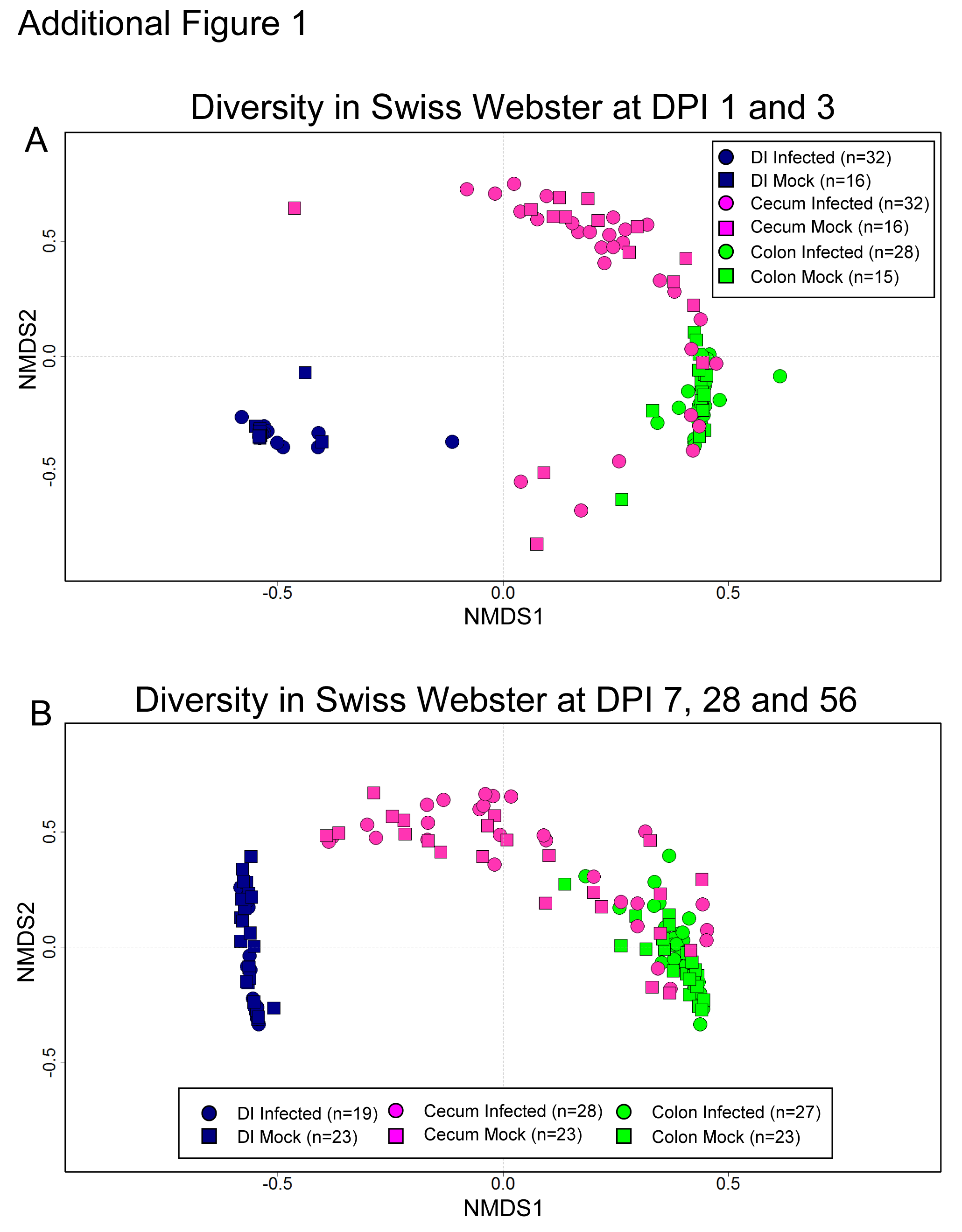

Supplement: Additional file 5: Figure S1 — Non-metric multidimensional scaling confirms communities are different by tissue site, but not by infection status, in Swiss Webster mice. This non-metric multidimensional scaling plot represents the relative operational taxonomic unit (OTU) abundance in Swiss Webster mice at a 3% definition level. (A) Community structure at early timepoints, 1 and 3 days post infection (DPI). (B) Community structure at late timepoints, 7, 28, and 56 DPI. Each murine intestinal community is represented by a symbol. Each symbol is colored to represent the tissue location of the community. All times were combined for each experiment. [file 2049-2618-1-7-S5.tiff]
